# Use of the Tissue Common Rejection Module Score in Kidney Transplant as an Objective Measure of Allograft Inflammation

**DOI:** 10.3389/fimmu.2020.614343

**Published:** 2021-02-03

**Authors:** Arya Zarinsefat, Jose M. Arreola Guerra, Tara Sigdel, Izabella Damm, Reuben Sarwal, Chitranon Chan-on, Gyula Szabo, Jorge L. Aguilar-Frasco, Xicohtencatl Ixtlapale-Carmona, Carlos Salinas-Ramos, Leonardo Ramirez-Martinez, Claudio Ramirez, Mario Vilatoba, Luis E. Morales Buenrostro, Josefina M. Alberu, Minnie M. Sarwal

**Affiliations:** ^1^ Department of Surgery, University of California, San Francisco, CA, United States; ^2^ Department of Internal Medicine, Centenario Hospital Miguel Hidalgo, Aguascalientes, Mexico; ^3^ Department of Pathology, University of California, San Francisco, CA, United States; ^4^ Instituto Nacional de Ciencias Medicas y Nutrición Salvador Zubirán, Mexico City, Mexico; ^5^ Department of Medicine, Tecnologico de Monterrey, Escuela de Medicina y Ciencias de la Salud, Mexico City, Mexico

**Keywords:** kidney transplant, acute rejection, biomarkers, transcriptomics, formalin-fixed paraffin-embedded (FFPE), graft inflammation

## Abstract

Long-term kidney transplant (KT) allograft outcomes have not improved as expected despite a better understanding of rejection and improved immunosuppression. Previous work had validated a computed rejection score, the tissue common rejection module (tCRM), measured by amplification-based assessment of 11 genes from formalin-fixed paraffin-embedded (FFPE) biopsy specimens, which allows for quantitative, unbiased assessment of immune injury. We applied tCRM in a prospective trial of 124 KT recipients, and contrasted assessment by tCRM and histology reads from 2 independent pathologists on protocol and cause biopsies post-transplant. Four 10-μm shaves from FFPE biopsy specimens were used for RNA extraction and amplification by qPCR of the 11 tCRM genes, from which the tCRM score was calculated. Biopsy diagnoses of either acute rejection (AR) or borderline rejection (BL) were considered to have inflammation present, while stable biopsies had no inflammation. Of the 77 biopsies that were read by both pathologists, a total of 40 mismatches in the diagnosis were present. The median tCRM scores for AR, BL, and stable diagnoses were 4.87, 1.85, and 1.27, respectively, with an overall significant difference among all histologic groups (Kruskal-Wallis, p < 0.0001*)*. There were significant differences in tCRM scores between pathologists both finding inflammation vs. disagreement (p = 0.003), and both finding inflammation vs. both finding no inflammation (p < 0.001), along with overall significance between all scores (Kruskal-Wallis, p < 0.001). A logistic regression model predicting graft inflammation using various clinical predictor variables and tCRM revealed the tCRM score as the only significant predictor of graft inflammation (OR: 1.90, 95% CI: 1.40–2.68, p < 0.0001). Accurate, quantitative, and unbiased assessment of rejection of the clinical sample is critical. Given the discrepant diagnoses between pathologists on the same samples, individuals could utilize the tCRM score as a tiebreaker in unclear situations. We propose that the tCRM quantitative score can provide unbiased quantification of graft inflammation, and its rapid evaluation by PCR on the FFPE shave can become a critical adjunct to help drive clinical decision making and immunosuppression delivery.

## Introduction

Kidney transplantation (KT) remains the preferred treatment for patients with end-stage renal disease, with reduced death rates compared to waitlist patients and improved survival among various demographic groups (1–3). Despite significant improvements in one-year kidney allograft survival (4), the rate of chronic graft loss following the first year post-transplant remains substantial. This has been observed despite a better understanding of allograft rejection, along with the advent of improved immunosuppression. Current classification methods of histological rejection suffer from sampling error and an inability to accurately quantify the inflammatory burden in the allograft, an important predictor of long-term graft function and survival (5–9). Importantly, prior studies have shown that pathologist correlation of Banff-graded renal allograft pathology have substantive discrepancies between biopsy interpretations (10, 11).

Previous work in our lab analyzing whole genome microarray data from 1,030 kidney, heart, lung, and liver allograft biopsies identified a common immune response module (CRM) of 11 genes that define acute rejection (AR) across varying allografts (12–14), and was subsequently validated on kidney allograft biopsies (15, 16). While this data demonstrated the potential utility of using an objective measure such as the CRM score to aid in biopsy diagnosis, these were retrospective analyses on previously banked tissue. Additionally, no comparisons were made between multiple blinded pathology reads of the same biopsy, or the potential ability of the CRM score to be used as an objective tiebreaker or ancillary data point to aid in arriving at a more robust diagnosis.

In this analysis, we looked at serial kidney biopsies that were obtained as part of a clinical trial in KT. We applied our protocol that measures gene expression of the 11 CRM genes from RNA isolated from four 10-micron shaves off a formalin-fixed paraffin embedded (FFPE) block with histologically confirmed phenotypes of AR, borderline rejection (BL), and stable diagnoses. We refer to this score as the tissue CRM (tCRM) given the application of the CRM score to biopsy tissue (15, 16). We then compared diagnoses from 2 blinded pathologists and compared them to the tCRM score for each biopsy. We hypothesized that the tCRM score could be used as a data point to aid in biopsy diagnosis, and possibly as an objective tiebreaker when there is disagreement among pathologists.

## Methods

### Patient Enrollment and Study Design

Patients and samples were collected as part of the TITRATE (Testing Immunosuppression Threshold in Renal Allografts To Extend eGFR) clinical trial. Its methodology can be consulted on the ClinicalTrials.gov portal (Identifier: NCT02581436). Briefly, this is a prospective, blinded, controlled clinical trial of 124 KT recipients who were assigned to two types of maneuvers in order to evaluate the efficacy and safety in terms of timely detection of AR, glomerular filtration rate (GFR), and fibrosis indices in protocol biopsies at 12 months post-KT. Monitoring of renal function was based on GFR and graft biopsies performed per protocol at 3 and 12 months post-KT and for cause. Induction immunosuppression (IS) was assigned based on immunological risk with Basiliximab (low-risk), anti-thymocyte globulin (high-risk) or without induction therapy in patients who shared two haplotypes. The maintenance IS was based on tacrolimus, mycophenolate, and prednisone.

The current study was an ancillary analysis for the tCRM genes (15) in all available KT biopsies. For our study, after excluding day zero biopsies, a total of 136 biopsies were available with matched biopsy histology, which were all interpreted by a pathologist at the National Institute of Medical Sciences in Mexico. Of these 136 biopsies, 77 were also sent to UCSF, to have blinded reads for further assessment of pathologist correlation. Biopsies were interpreted based on the most recent Banff criteria at the time of the study (17). Any form of graft AR (acute cellular or antibody-mediated) was combined into one AR category. For the UCSF-read biopsies, more granular histopathologic diagnosis data was available, including antibody-mediated rejection (ABMR) type, and T-cell mediated rejection (TCMR) type.

### Total RNA Extraction

We followed previously published protocol for the extraction of RNA from FFPE tissues (18). Based on our previous experience, we used 4 × 10 μm-thick sections to extract total RNA from FFPE samples with the PureLink FFPE Total RNA Isolation it (Thermo Fisher Scientific, Foster City, CA). The RNA data quality was assessed by 260/280 absorption signal ratio and the RIN number.

### cDNA Synthesis and Gene Expression Quantification Using qPCR

A total of 50 ng RNA was reversed transcribed into complementary DNA (cDNA) using SuperScript VILO (Invitrogen, Thermo Fisher Scientific, Foster City, CA) and then amplified in a target specific amplification step for all 11 genes using TaqMan PreAmp Master Mix and TaqMan Primers and Probes (Thermo Fisher Scientific, Foster City, CA) for a total of 18 amplification cycles. qPCR reactions were performed in the Fluidigm BioMark FD system (Fluidigm, South San Francisco, CA) using an 18S gene housekeeping gene and Human XpressRef Universal Total RNA (Qiagen, Valencia, CA) as a reference RNA for 40 cycles. Resulting chip data was initially analyzed for quality control using the BioMark Analysis Software Version 2.0 (Fluidigm, South San Francisco, CA) and Ct values were exported into a spreadsheet. Normalization of the data was done in two steps. Ct values of individual genes were normalized against Ct value of 18S for each gene to get dCt values. dCt values of each sample were normalized against dCt values of the reference sample to get ddCt values which were subsequently used to calculate fold change (RQ) values for each gene in each sample. The tCRM score was calculated by taking the geometric mean of the 11 CRM genes for each sample, as previously described (12).

### Statistical Analysis

All data was imported into *R* version 3.6.2 (R Foundation, Vienna, Austria) for subsequent analyses. Patient demographics (**Table 1**) are shown based on tCRM score greater than or less than 2.2, our previously determined cutoff with optimal AUROC for predicting AR (15). Statistical testing was performed with Student’s t-test for continuous variables and chi-square test for categorical variables. Bubble and bar plots of the tCRM score for each pathologic diagnosis group were made using the *ggplot2* package. Significant differences between all pathologic groups and pairwise differences between each group were determined by the Kruskal-Wallis test. Unsupervised hierarchical clustering utilizing the different pathologic groups and tCRM genes was performed, with subsequent heatmaps made using the *pheatmap* package. We considered tCRM scores greater than 2.2 to have a molecular diagnosis of rejection. We defined a biopsy as being positive for inflammation if the pathologic diagnosis was either AR or BL, and negative for inflammation if the diagnosis was stable. We then created an agreement variable between pathologists, where both stated inflammation was present, both said there was no inflammation, or they disagreed if any inflammation was present. Bar plots and heatmaps for the tCRM scores among the agreement variable were then created as described above.

**Table 1:** Patient demographics.

	tCRM < 2.2 (n = 49)	tCRM > 2.2 (n = 28)	P-value
Age, mean (SD)	36.0 (12.8)	40.5 (11.1)	0.11
BMI, mean (SD)	23.1 (4.0)	24.1 (4.2)	0.29
Cause, no. (%)			0.23
Diabetes	10 (20)	2 (7)	
Glomerulonephritis	1 (2)	0 (0)	
Hypertension	5 (10)	2 (7)	
Other	8 (16)	10 (36)	
Unknown	25 (51)	14 (50)	
DGF, no. (%)	2 (4)	4 (14)	0.24
Donor age, mean (SD)	36.2 (13)	45.9 (13)	0.001
Donor gender: male, no. (%)	25 (51)	14 (50)	1.0
Gender: male, no. (%)	28 (57)	9 (32)	0.06
Induction, no. (%)			0.40
Basiliximab	12 (24)	8 (28)	
Thymoglobulin	34 (69)	20 (71)	
Other	3 (6)	0 (0)	
Living donor, no. (%)	24 (49)	9 (32)	0.23
Pre-transplant dialysis, no. (%)	42 (86)	23 (82)	0.93
Previous transplant, no. (%)	3 (7)	3 (11)	0.78

The patient demographics were compared based on whether the tCRM score was greater than or less than 2.2. The only significant difference between these groups relates to donor age. Bolded p-values indicate a significant p-value < 0.05.

We computed a logistic regression model to predict the binary outcome of inflammation on biopsy. We input recipient age, body mass index (BMI), delayed graft function (DGF), gender, induction regimen, living donor status, male donor to female recipient, previous KT, pre-KT dialysis, and the tCRM score as predictor variables in the model.

**Figure 1:**
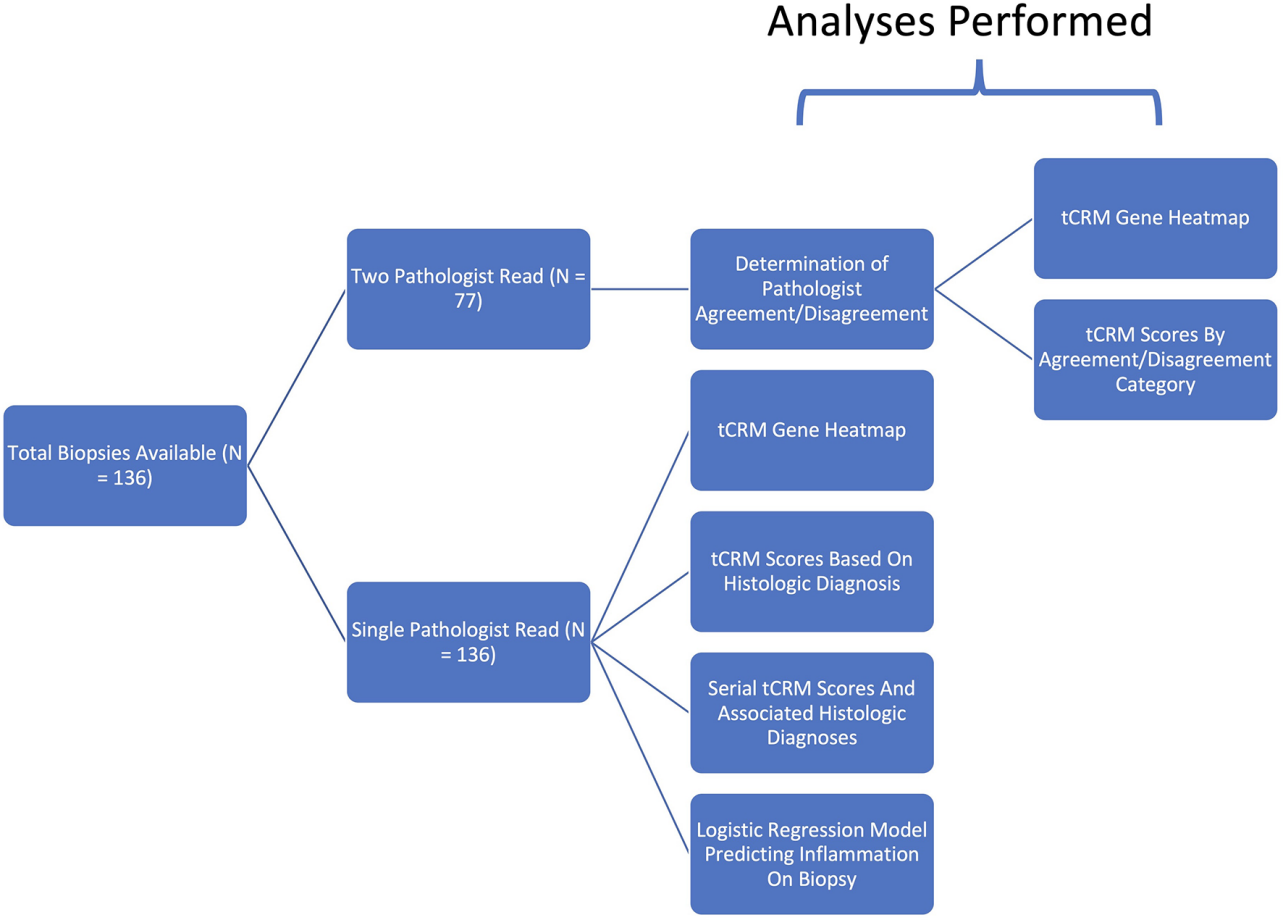
Schematic of study design and analyses performed.

We obtained each patient’s delta GFR, calculated as the change in their time of biopsy GFR from their final GFR at two years post-KT. We used the Pearson correlation coefficient to correlate the delta GFR to the patient’s tCRM score that was obtained from the biopsy. This was repeated after excluding tCRM scores less than 2. We utilized the highest donor-specific antibody (DSA) levels that were measured by flow cytometry for each patient, represented as mean fluorescence intensity (MFI), to perform correlation to tCRM scores and calculate the associated Pearson correlation coefficient. This correlation was also presented in scatter plot format. For analysis of serial scores, we plotted patients with serial tCRM scores by post-transplant days, focusing on the four patients that had an original diagnosis of AR, were treated, and were then followed with a subsequent biopsy. Two additional patients that had elevated tCRM scores which were read as having a stable diagnosis, but then developed a new diagnosis of AR on their follow up biopsies were also plotted. Study design and subsequent analysis is further summarized in a pictorial form (**Figure 1**).

## Results

### Kidney Allograft Biopsy Findings from Two Independent, Blinded Pathologists

Patient demographics and clinical variables of interest showed no major differences between patients that had tCRM scores greater than or less than 2.2, except for higher donor age in patients with elevated tCRM scores on post-transplant biopsies (**Table 1**). A total of 77 biopsies were read by both centers, with pathologist 1 (Mexico) diagnosing 16 AR, 21 BL, and 40 stable biopsies, while pathologist 2 (UCSF) diagnosed 10 AR, 39 BL, and 28 stable biopsies (**Table 2**). There were a total of 40 mismatched diagnoses between the pathologists, with the bulk of these mismatches coming from differences in the BL category.

**Table 2:** Histologic diagnoses per pathologist.

Site Specific Histology Reads	Rejection	Borderline Rejection	Stable	Total
Mexico	16	21	40	77
UCSF	10	39	28	77

The individual diagnoses for each pathologist at each center is shown. Overall 77 biopsies were available to both pathologists to be read. A total of 40 mismatches between pathologists was found, with the most notable discrepancy between pathologists occurring in the borderline rejection group.

### Tissue Common Rejection Module as a Tiebreaker Discriminates Between Pathologic Diagnoses

**Figure 2:**
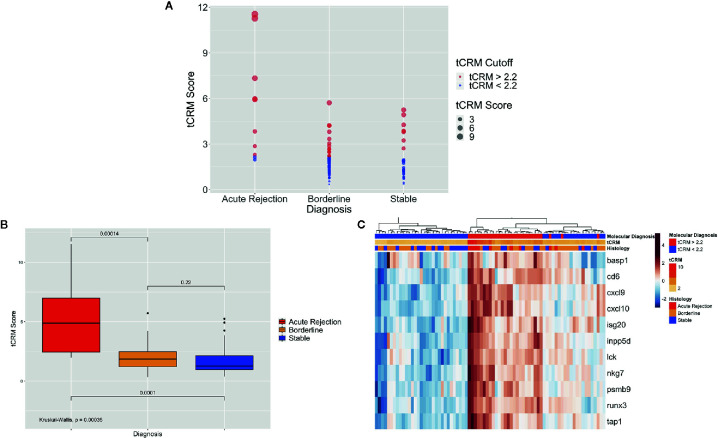
Bubble and bar plots of tCRM scores and heatmap of tCRM genes from individual pathologist diagnoses. **(A)** Bubble plot of tCRM scores by pathologic diagnosis, with increasing diameter of bubbles indicating higher tCRM scores; colored by tCRM cutoff of 2.2, with red greater than 2.2, and blue less than 2.2; this shows a distribution of molecular (tCRM) scores that do not always follow histology classification. **(B)** Box plot of tCRM scores by pathologic diagnosis; differences in tCRM scores by histologic groups shown by overall Kruskal-Wallis test and pairwise comparisons between the three categories with P-values shown, with overall significance between all groups and pairwise comparisons. **(C)** Heatmap with unsupervised hierarchical clustering of the 11 tCRM genes, annotated by tCRM cutoff less than or greater than 2.2 (molecular diagnosis), color gradient representing actual tCRM score, and histologic diagnosis by pathologist; overall biopsies shown to cluster with higher tCRM scores seen in histologic diagnoses with inflammation present.

We then looked at the ability of the tCRM score to discriminate between pathologic diagnoses for a single pathologist (utilizing biopsies read at UCSF, given that we had the most detailed histopathologic data available). When looking at the tCRM scores by histologic diagnosis, we saw a clear separation with higher tCRM scores in patients with AR compared to BL/stable, and with a higher proportion of scores > 2.2 in AR patients (**Figure 2A**). The median tCRM scores and interquartile ranges (IQR) for AR, BL, and stable diagnoses were 4.87 (IQR: 2.44–6.99), 1.85 (IQR: 1.21–2.48), and 1.27 (IQR: 0.96–2.14), respectively. Additionally, there was an overall significant difference among all histologic groups (p < 0.001*)*, along with significant differences among AR vs. BL and AR vs. stable pairwise comparisons (**Figure 2B**). This highlights the correlation of tCRM scores with Banff-graded pathology.

When performing unsupervised hierarchical clustering of the tCRM genes, we see relatively accurate clustering by the pathologic diagnosis (**Figure 2C**). When examining each biopsy closely, even where clustering by diagnosis shows heterogeneity in the clustered groups, the corresponding tCRM score (shown as a color gradient) tends to correlate with the diagnosis. We see higher scores with diagnoses reflective of greater levels of inflammation, signifying the ability of tCRM to discriminate and objectively quantify inflammation in the allograft without bias. When we utilized our tCRM cutoff of 2.2 (molecular diagnosis of inflammation), we see strong clustering by molecular diagnosis. Regardless, the majority of AR and BL cluster together with high gene expression and high tCRM scores, with the opposite for most histologically-graded stable biopsies.

### Tissue Common Rejection Module Discriminates between Specific Types of Graft Rejection

**Figure 3:**
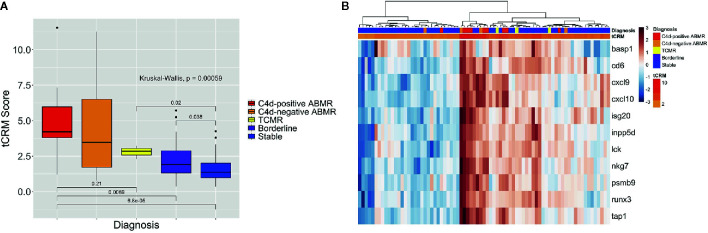
Box plot of tCRM scores and heatmap for all rejection types. **(A)** Box plot of tCRM scores by pathologic diagnosis; differences in tCRM by histologic groups shown by overall Kruskal-Wallis test and pairwise comparisons between C4d-positive ABMR vs. TCMR, C4d-positive ABMR vs. BL, C4d-positive ABMR vs. stable, TCMR vs. stable, and BL vs. stable categories. **(B)** Heatmap with unsupervised hierarchical clustering of the 11 tCRM genes, annotated by color gradient representing actual tCRM score, and histologic diagnoses.

We then looked at the ability of the tCRM score to distinguish between different types of rejection: C4d-positive (n = 9) and negative ABMR (n = 4), TCMR (n = 3), BL (n = 21), and stable (n = 40) biopsies. The median tCRM scores and IQR for C4d-positive ABMR, C4d-negative ABMR, TCMR, BL, and stable diagnoses were 4.22 (IQR: 3.80–5.98), 3.47 (IQR: 1.71–6.51), 2.86 (IQR: 2.58–3.05), 1.91 (IQR: 1.32–2.88), and 1.37 (IQR: 0.99–2.00), respectively. Additionally, there was an overall significant difference among all histologic groups (p < 0.001*)*, along with significant differences among pairwise comparisons looking at C4d-positive ABMR vs. TCMR, C4d-positive ABMR vs. BL, C4d-positive ABMR vs. stable, TCMR vs. stable, and BL vs. stable groups (**Figure 3A**). This once again highlights the correlation of tCRM scores with specific Banff-graded pathology.

When performing unsupervised hierarchical clustering of the tCRM genes, we see similar patterns of clustering based on graft inflammation as previously described (**Figure 3B**). With the more specific diagnostic categories of C4d-positive and negative ABMR and TCMR, we do see the most notable homogenous clustering among ABMR groups. As before, when examining each biopsy closely, even where clustering by diagnosis shows heterogeneity in the clustered groups, the corresponding tCRM score (shown as a color gradient) tends to correlate with the diagnosis. We see higher scores with diagnoses reflective of greater levels of inflammation, signifying the ability of tCRM to discriminate and objectively quantify inflammation in the allograft without bias. Ultimately, the first branch of our unsupervised clustering contained the majority of samples that had any degree of graft inflammation, specifically any ABMR, TCMR, or BL cases (**Figure 3B**).

### Tissue Common Rejection Module Discriminates by Pathologist Agreement/Disagreement

**Figure 4:**
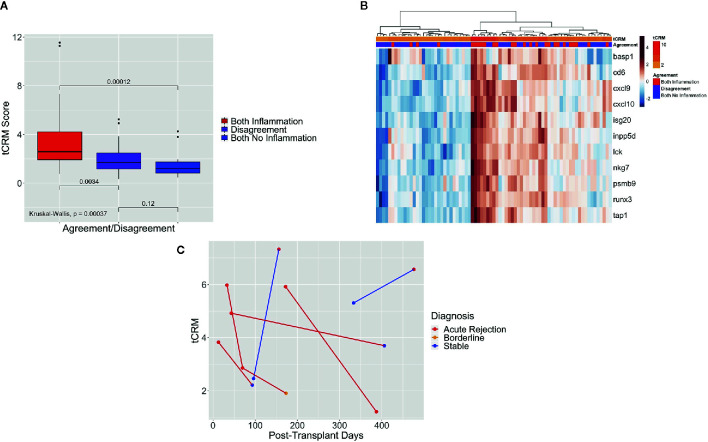
Box plot of tCRM scores of pathologist agreement, heatmap of tCRM genes based on pathologist agreement, and serial tCRM scores. **(A)** Box plot of tCRM scores by pathologist agreement/disagreement; differences in tCRM scores by agreement/disagreement category shown by overall Kruskal-Wallis test and pairwise comparisons between the three categories with P-values shown, with overall significance between all groups. **(B)** Heatmap with unsupervised hierarchical clustering of the 11 tCRM genes, annotated by the agreement variable and color gradient representing tCRM score; clustering and tCRM score distribution shows correlation between higher tCRM scores and both pathologists agreeing upon presence of inflammation, with lower tCRM scores correlating to both pathologists agreeing upon the absence of inflammation. **(C)** Scatter plot of serial tCRM scores by post-transplant days, showing scores for the four patients with elevated tCRM scores and pathologic diagnosis of acute rejection (individual patient’s serial scores connected with red lines), with subsequent decrease of serial tCRM scores after treatment; blue lines mark the two patients with elevated tCRM scores but diagnosis of stable biopsy, and subsequent biopsy showing rejection and continued elevation of tCRM score.

We then looked at the tCRM scores for agreement and disagreement among pathologists, which was defined as previously explained. Of the 77 post-transplant biopsies that were read by both pathologists, both said there was inflammation in 33.8%, no inflammation in 22.1%, and disagreed upon the presence of inflammation in 44.2% of samples. When examining the tCRM scores, we noted an increase in scores as the agreement variable progressed from no inflammation, disagreement, and agreement upon the presence of inflammation (**Figure 4A**). There were significant differences in tCRM scores between pathologists both finding inflammation vs. disagreement (p = 0.003), and both finding inflammation vs. both finding no inflammation (p < 0.001), along with overall significance between all scores (p < 0.001).

When performing unsupervised hierarchical clustering of the tCRM genes, we saw clustering of biopsy samples based on the agreement variable (**Figure 4B**). We can see that the clusters of high gene expression correspond to both pathologists agreeing upon the presence of inflammation, while in contrast, low gene expression corresponds to both pathologists agreeing upon no inflammation or disagreement between them. Additionally, we see that the corresponding tCRM score for each biopsy tends to favor higher and lower scores where pathologists agreed on inflammation or no inflammation, respectively. While the clustering is not perfect, a clear trend is visible.

### Tissue Common Rejection Module as a Predictor of Rejection and Graft Function

Above, we show the clinical utility of tCRM as it relates to making accurate pathologic diagnoses. Although its application there relates to what is traditionally a biopsy-proven diagnosis, we wanted to explore tCRM’s use in a model for predicting rejection/inflammation. Using our binary variable of inflammation (AR and BL biopsies), we created a logistic regression model to predict the binary outcome of inflammation on biopsy. The clinical variables used were previously described (**Table 3**). Interestingly, the only significant predictor of inflammation as diagnosed by pathology was the tCRM score (OR: 1.90, 95% CI: 1.40–2.68, p < 0.001), while having a male donor to female recipient was protective with borderline significance (OR: 0.27, 95% CI: 0.07–0.98, p = 0.05).

**Table 3:** Logistic regression model for graft inflammation.

	Adjusted Odds Ratio (95% CI)	P-value
Age	1.00 (0.96–1.04)	0.96
BMI	1.03 (0.90–1.17)	0.70
DGF	2.61 (0.38–22.5)	0.34
Gender (Male)	0.58 (0.19–1.76)	0.34
Induction		
Basiliximab	0.71 (0.08–16.4)	0.79
Thymoglobulin	1.14 (0.11–27.4)	0.92
Living Donor		
Living-Related	0.42 (0.10–1.68)	0.23
Living-Unrelated	0.27 (0.05–1.15)	0.09
Male Donor To Female Recipient	0.27 (0.07–0.98)	0.05
Previous Transplant	1.54 (0.28–7.68)	0.60
Pre-Transplant Dialysis	0.51 (0.12–2.21)	0.35
tCRM	1.90 (1.40–2.68)	**<0.001**

The predictor variables that were input into the logistic regression model for prediction of graft inflammation is shown. Graft inflammation was defined as either an acute or borderline rejection diagnosis. Only the tCRM score was found to be a significant predictor of graft inflammation on biopsy. Bolded p-values indicate a significant p-value < 0.05.

We then looked at the ability of tCRM to predict long-term changes in GFR. We correlated the delta GFR (from the GFR at the time of biopsy to the final GFR) to the patient’s tCRM score from that biopsy (Pearson correlation coefficient = 0.21, p = 0.025). We then repeated this after excluding tCRM scores less than 2, so that we were only looking at the most elevated tCRM values to see the effect on delta GFR, which resulted in an increase in correlation coefficient to 0.32 (p = 0.05). We also looked at the correlation of each patient’s highest DSA level with their associated tCRM score (**Supplementary Figure 1**), to see if there was any association between DSA levels and tCRM scores, which we did not find to be correlated (Pearson correlation coefficient = 0, p = 0.99).

Finally, we looked at patients that had serial biopsies and tCRM scores. Serial tCRM scores were plotted per patient by post-transplant days (**Figure 4C**). There were four patients that had an original diagnosis of AR and were treated, then followed with a subsequent biopsy (**Figure 4C**, red lines). We noted that all four patients initially had an elevated tCRM score, and post-treatment had a decrease in their score, with only one patient being diagnosed with AR on the second biopsy. Of note, two patients that had elevated tCRM scores which were read by the pathologist as having a stable diagnosis, developed an increase in their tCRM score and a new diagnosis of AR on their follow up biopsies (**Figure 4C**, blue lines).

## Discussion

We hypothesized that the tCRM score could be used as a data point to aid in biopsy diagnosis, and possibly as an objective tiebreaker when there is disagreement among pathologists. Above we show that the tCRM score can discriminate between pathologic diagnoses. tCRM is able to discriminate between combined AR vs. BL vs. stable samples, and is also able to discriminate between more specific AR diagnoses such as ABMR vs. TCMR and ABMR vs. BL, as we demonstrated above. One of the known drawbacks of the combined subjective/objective nature of the Banff criteria is that discrepancies can exist between pathologists looking at identical biopsy specimens. We demonstrate above that disagreement between pathologists regarding the inflammatory burden in kidney biopsies is common. We show that when there is agreement among pathologists on the presence or absence of inflammation, this tends to be at the extremes of the tCRM score. Part of the utility in the tCRM score may lie here, in its ability to provide an object measure of inflammation, but also serve as a tiebreaker in cases where the diagnosis is not clear, but the tCRM score clearly points one way or another. For example, a pathologist reading a biopsy that is not certain of classifying it as AR or BL, but with a very high tCRM score, could be more objective and accurate about making the diagnosis of AR.

In addition, this study indicates that the tCRM score may be used to predict more long-term clinical outcomes. Although the correlation between changes in GFR and higher tCRM scores is weak, we showed a statistically significant positive correlation between them, with an even greater correlation when we filtered for tCRM scores greater than 2, suggesting that more elevated scores suggest a greater degree of allograft injury which can predict long-term reduced graft function (greater delta GFR). Interestingly, we did not find a correlation between patient DSA levels and tCRM scores. This may partly be due to the fact that many patients with DSAs do not necessarily go on to develop ABMR, along with knowing that different DSAs may confer differing risks of actually developing ABMR. Additionally, by tracking patients with serial biopsies and tCRM scores, we showed that the majority of patients with elevated tCRM scores and AR had a subsequent decrease in their scores, along with improvement in graft inflammation on subsequent biopsies (subsequent histology went from AR to stable). Notably, there were 2 cases where the tCRM scores were markedly elevated, yet the pathologic diagnosis was stable on biopsy. Both of these cases resulted in an even more elevated tCRM score on the subsequent biopsy, with a corresponding pathologic diagnosis of AR. This suggests the possibility of either misdiagnosis due to the subjective nature of histologic classification, or that the elevated expression of the tCRM genes that correlate with graft inflammation, may predate actual pathologic findings that would result in a diagnosis of AR. Regardless, this suggests the utility of the more objective tCRM score in helping make the diagnosis of AR.

Molecular quantification of the overall inflammatory burden in the renal allograft is important to establish at the time of an invasive biopsy (5, 6, 9). Our method facilitates the use of already available biopsy specimens in an efficient manner (overall experiment times are 4–6 h for the assays shown). While non-invasive tests, such as donor-derived cell-free DNA (dd-cfDNA), are being proposed for the early diagnosis of AR (19–23), one drawback of these tests is that they do not directly interrogate the target tissue of interest, even if the dd-cfDNA is released from the allograft. With tCRM, biopsy tissue that is already being obtained can be processed using minimal tissue from either fresh biopsies or archived FFPE blocks, preserving most of the parent FFPE block for additional histologic analyses.

Our study is partly limited by the invasive nature of tCRM. Biopsies remain the gold standard and are frequently obtained, allowing for molecular profiling with tCRM, but ultimately, a transition to non-invasive tests on the blood or urine would be most ideal for patients. Our lab is currently working on the use of the CRM genes in urine to aid in the diagnosis of graft rejection (14). Additionally, while this study was a prospective trial, the application of tCRM was not used for clinical decision making, and was instead an exploratory analysis. Our future work would benefit from considering the tCRM score as a possible endpoint, or actually factoring in the score when deciding on the final pathologic diagnosis for a biopsy specimen. Additionally, while our study samples included TCMR diagnoses, the majority of our available specimens for analysis were ABMR specimens. While we show that the tCRM score is elevated regardless of the type of AR present, future work will need to utilize greater numbers of specimens within each category of specific AR type.

We showed that the tCRM score is able to discriminate between pathologic diagnoses, using already available FFPE specimens. Importantly, variability exists among different pathologists, which was demonstrated in our data based on two blinded pathologists making diagnoses. As such, we demonstrate that not only can tCRM aid in pathologic diagnosis, but especially when disagreement or uncertainty exists, the score may be utilized to reach a final, more robust conclusion.

## Data Availability Statement

The raw data supporting the conclusions of this article will be made available by the authors, without undue reservation.

## Ethics Statement

The studies involving human participants were reviewed and approved by UCSF IRB. The patients/participants provided their written informed consent to participate in this study.

## Author Contributions

AZ contributed to the analysis design, data analysis, interpretation, manuscript writing, and manuscript editing. JG contributed to the study design, data collection, and manuscript review. TS contributed to the data analysis and manuscript editing. ID contributed to the sample processing. RS, and GS contributed to the sample processing. CC contributed to the sample processing and review. JF, XC, CSR, LM, and CR contributed to sample and database collection. MV contributed to the patient enrollment and transplantation. LB contributed to the patient enrollment. JA contributed to the study design, data collection, and manuscript review. MS contributed to the study design, data collection, analysis design, and manuscript review. All authors contributed to the article and approved the submitted version.

## Funding

AZ was funded by the NIH: 5 T32 AI 125222.

## Conflict of Interest

The authors declare that the research was conducted in the absence of any commercial or financial relationships that could be construed as a potential conflict of interest.

## Supplementary Material

Supplementary Figure 1 Scatter plot of donor-specific antibody levels and tCRM scores. Scatter plot with x-axis representing donor-specific antibody (DSA) levels represented by mean fluorescence intensity (MFI) and y-axis representing the corresponding tCRM scores from that specific patient’s biopsy; Pearson correlation coefficient = 0 with a corresponding p-value of 0.99.
