# Supplementary figures and images for: Use of the Tissue Common Rejection Module Score in Kidney Transplant as an Objective Measure of Allograft Inflammation
Source: Front Immunol. 2021 Feb 3;11:614343. doi: 10.3389/fimmu.2020.614343 (PMC7886808; doi:10.3389/fimmu.2020.614343)

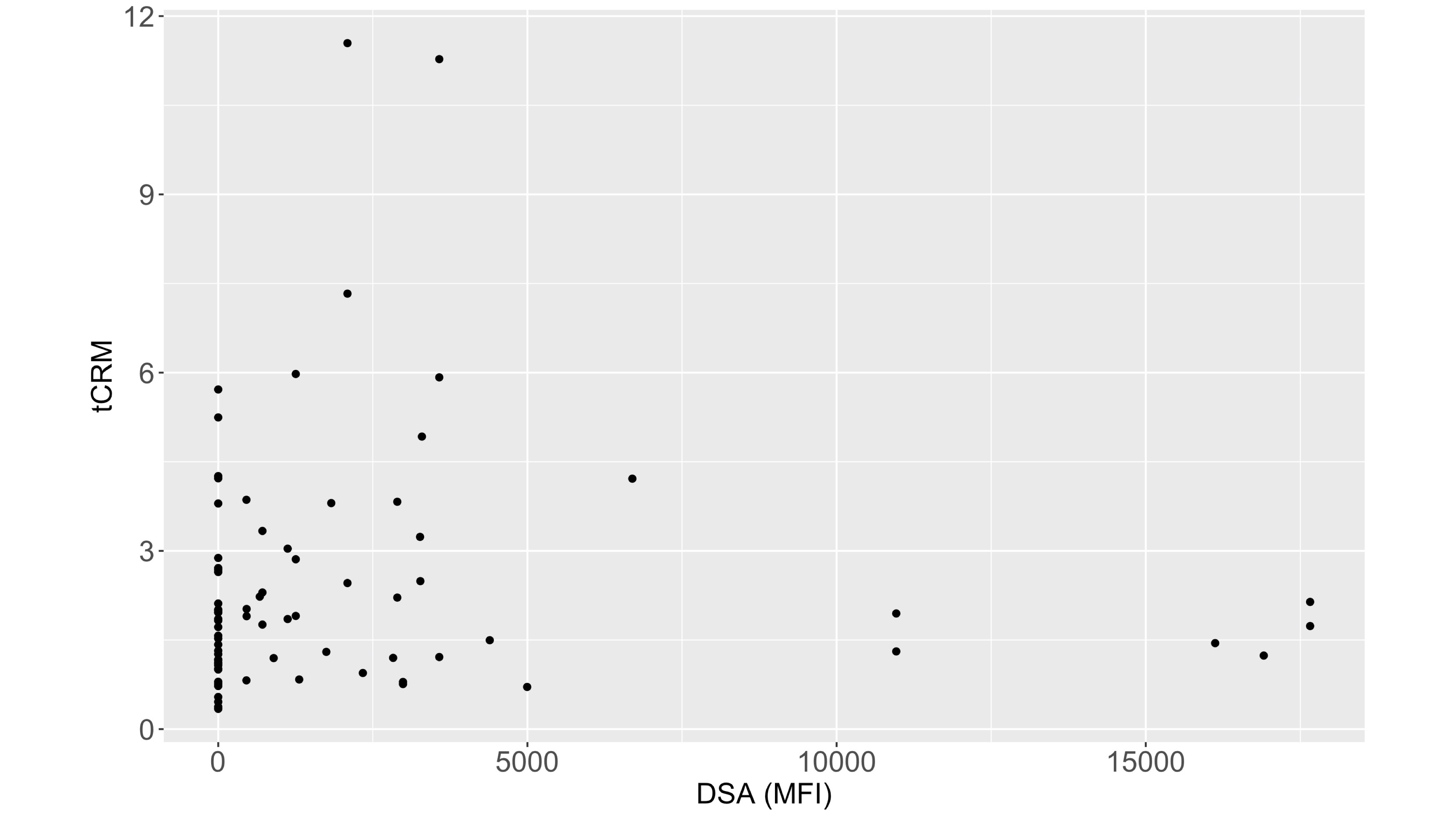

Supplement: Supplementary Figure 1 — Scatter plot of donor-specific antibody levels and tCRM scores. Scatter plot with x-axis representing donor-specific antibody (DSA) levels represented by mean fluorescence intensity (MFI) and y-axis representing the corresponding tCRM scores from that specific patient’s biopsy; Pearson correlation coefficient = 0 with a corresponding p-value of 0.99. [file Image_1.jpeg]
